# Ancestral polymorphism at the major histocompatibility complex (MHCIIß) in the *Nesospiza* bunting species complex and its sister species (*Rowettia goughensis*)

**DOI:** 10.1186/1471-2148-12-143

**Published:** 2012-08-15

**Authors:** Alexandra Jansen van Rensburg, Paulette Bloomer, Peter G Ryan, Bengt Hansson

**Affiliations:** 1 Molecular Ecology and Evolution Program, Department of Genetics, University of Pretoria, Private bag X20, Hatfield, 0028, South Africa; 2 Percy FitzPatrick Institute of African Ornithology, DST/NRF Centre of Excellence, University of Cape Town, Rondebosch, 7701, South Africa; 3 Molecular Ecology and Evolution Lab, Department of Biology, Lund University, SE-22362, Lund, Sweden

## Abstract

**Background:**

The major histocompatibility complex (MHC) is an important component of the vertebrate immune system and is frequently used to characterise adaptive variation in wild populations due to its co-evolution with pathogens. Passerine birds have an exceptionally diverse MHC with multiple gene copies and large numbers of alleles compared to other avian taxa. The *Nesospiza* bunting species complex (two species on Nightingale Island; one species with three sub-species on Inaccessible Island) represents a rapid adaptive radiation at a small, isolated archipelago, and is thus an excellent model for the study of adaptation and speciation. In this first study of MHC in *Nesospiza* buntings, we aim to characterize MHCIIß variation, determine the strength of selection acting at this gene region and assess the level of shared polymorphism between the *Nesospiza* species complex and its putative sister taxon, *Rowettia goughensis,* from Gough Island.

**Results:**

In total, 23 unique alleles were found in 14 *Nesospiza* and 2 *R. goughensis* individuals encoding at least four presumably functional loci and two pseudogenes. There was no evidence of ongoing selection on the peptide binding region (PBR). Of the 23 alleles, 15 were found on both the islands inhabited by *Nesospiza* species, and seven in both *Nesospiza* and *Rowettia*; indications of shared, ancestral polymorphism. A gene tree of *Nesospiza* MHCIIß alleles with several other passerine birds shows three highly supported *Nesospiza*-specific groups. All *R. goughensis* alleles were shared with *Nesospiza*, and these alleles were found in all three *Nesospiza* sequence groups in the gene tree, suggesting that most of the observed variation predates their phylogenetic split.

**Conclusions:**

Lack of evidence of selection on the PBR, together with shared polymorphism across the gene tree, suggests that population variation of MHCIIß among *Nesospiza* and *Rowettia* is due to ancestral polymorphism rather than local selective forces. Weak or no selection pressure could be attributed to low parasite load at these isolated Atlantic islands. The deep divergence between the highly supported *Nesospiza*-specific sequence Groups 2 and 3, and the clustering of Group 3 close to the distantly related passerines, provide strong support for preserved ancestral polymorphism, and present evidence of one of the rare cases of extensive ancestral polymorphism in birds.

## Background

Understanding the principals that govern the generation and maintenance of functional genetic diversity is fundamental to evolutionary biology. Large reductions in population size, through bottleneck or founder events, result in a loss of genetic diversity [1] which may affect the ability of populations to adapt and survive in changing environments [1,2]. However, genes of ecological adaptive importance may maintain variation through a severe reduction in population size through processes such as balancing selection [3,4]. The Major Histocompatibility Complex (MHC) is such a functional locus, and has been extensively studied in both model and non-model species [5-7].

The MHC is a multigene family involved in the vertebrate immune response [8], and is the most polymorphic set of genes known in vertebrates [9,10]. MHC variation is driven by an arms race between host and pathogen, where balancing selection maintains alleles in the population. An extensive repertoire of alleles enables the population to respond rapidly to changing or novel pathogens [11-13]. The highly variable peptide binding region (PBR) encoded by MHC class II ß exon 2 (MHCIIß) ensures the binding of a large number of conformationally different peptides [8]. The PBR of MHC molecules is involved in antigen recognition and as such may be under strong balancing selection when compared with the non-PBR sites [14]. Although the major driving force behind MHC diversity is host-pathogen co-evolution [11,15], sexual selection and selection against deleterious mutations also play a role in the maintenance of MHC variation [16-18].

Like many multi-gene families, MHC is governed by the birth-and-death model of evolution where new genes are generated through gene duplication. Some of these genes are maintained for long periods and even through population divergence events, while others lose function (pseudogenes) or are lost completely. MHC variation is also governed by gene conversion, where homologous recombination occurs between duplicated genes (paralogous genes), thus homogenising sequences between different loci [6,19]. In passerine birds, the MHC is characterised by multiple gene copies, pseudogenes and long introns, and is exceptionally diverse and complex compared to other birds and vertebrate species [20-22]. Gene duplication events of MHC can be traced phylogenetically in most lineages, because duplicated genes evolve independently. This can be seen in the phylogenetic grouping of orthologous genes, rather than in a species-specific grouping [19,23,24]. Alternatively, recent duplication and concerted evolution of genes (through gene conversion) can result in species-specific clustering [6,22,25,26]. Due to the high rate of gene duplication and loss, and the confounding effect of gene conversion, it is notoriously difficult to re-construct avian MHC phylogenies [6].

Following a bottleneck or founder event, the genetic diversity of a population is reduced to only a subset of the original variation. As the population adapts to its new environment, the MHC allelic diversity will be made up of a combination of ancestral polymorphism and novel genetic variation. Trans-species evolution [27] or ancestral polymorphism [28] refers to the long-term maintenance of ancestral alleles in populations and species [29,30]. This process is governed by balancing selection [31] and is seen when related species or subspecies share similar or the same MHC alleles despite local selection pressure. This pattern is common in mammals which do not often show concerted evolution, thus orthologous loci can be recognized between distantly related taxa such as mice and humans [24]. The high levels of concerted evolution in birds often make it difficult to distinguish between orthologous and paralogous loci [25], although isolated cases have been reported e.g. [5,32]. Novel genetic diversity is introduced in populations either through dispersal or mutations. Mutational processes include gene duplication, point mutations and gene conversion e.g. [26,33]. Gene conversion is known to occur frequently in birds at the highly duplicated MHC genes [6,26,34,35]. The rate of gene conversion has been shown to be far greater than that of point mutations, thus may be a very important mechanism for generation of variation in bottlenecked populations [9,26].

In the present study, we assess MHC variation in the *Nesospiza* bunting species complex and its putative sister taxon, *Rowettia goughensis*. Evaluation of the MHC in *Nesospiza* and *R. goughensis* is interesting for several reasons. *Nesospiza* and *R. goughensis* are considered sister taxa and are presumed to have arrived at Tristan da Cunha and nearby Gough Island with the same colonization event [36]. Mitochondrial *cytochrome b* sequences are reciprocally monophyletic between island systems, and neutral microsatellite markers show substantial genetic differentiation between species [37,38]. It is thus interesting to compare the MHC differentiation and allele sharing in *Nesospiza* and *R. goughensis* and determine the level of ancestral polymorphism between these species. Further, *Nesospiza* buntings have undergone an ecological adaptive radiation in parallel on two islands [37]. Both Nightingale and Inaccessible islands are inhabited by large- and small-billed *Nesospiza* buntings. The two species on Nightingale Island (*N. questi and N. wilkinsi*) co-occur with little, if any, interbreeding, probably due to the availability of two discrete seed sizes within a single habitat. Inaccessible Island has three lineages of *N. acunhae* buntings: large-billed *N. a. dunnei*, and two colour morphs of the small-billed bunting, *N. a. fraseri* and *N. a. acunhae*[37,39]. Hybridisation occurs between all three forms across an ecotone on the eastern plateau of Inaccessible Island. This is probably due to a large variation of seed sizes occurring at low densities, which favours greater diversity in bill-sizes [37]. A single *Nesospiza* species inhabited the main island of Tristan, but was driven to extinction shortly after the arrival of humans at the archipelago. Genetic structure analysis based on neutral microsatellite markers show little or no hybridization between species on Nightingale, and strong differentiation between Nightingale *Nesospiza* and those on Inaccessible Island [37,38]. Despite ongoing hybridization on Inaccessible Island, a strong association has been found between bill morphology, habitat choice and genetic differentiation suggesting that both natural and sexual selection may maintain differentiation [37,38]. Thus, it is possible that these selective pressures will result in species-specific patterns of MHC variation. However, an alternative hypothesis is that balancing selection has maintained most of the MHC variation across the species complex. Here we aim to 1) test for signatures of selection at the MHCIIß in *Nesospiza* buntings, and 2) investigate the extent of ancestral polymorphism between *Nesospiza,* its putative sister taxon *Rowettia goughensis*, and other passerine species [5,32,34,35,40,41].

## Results

### PCR amplification success and nucleotide diversity

**Figure 1:**
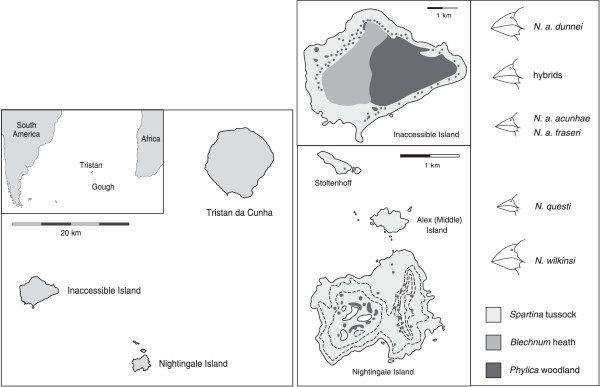
**Geographic location, vegetational composition, and *Nesospiza *populations occurring at the Tristan da Cunha archipelago.** Location of the Tristan da Cunha archipelago in the South Atlantic Ocean with the three main islands: Tristan, Inaccessible, and Nightingale. The vegetational composition, and occurring species and morpho-types of *Nesospiza* buntings are shown for Inaccessible and Nightingale islands (adapted from reference 37 and Google Maps).

**Figure 2:**
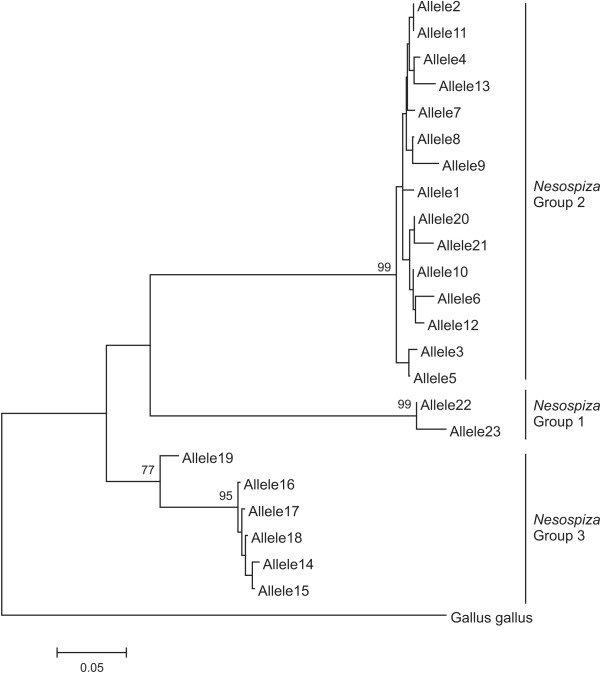
***Nesospiza *MHCIIβ gene tree.** Neighbour-Joining tree showing the three well supported *Nesospiza* MHCIIβ exon 2 allele clusters. Of the 23 alleles, 21 were found in the *N. acunhae* individuals on Inaccessible Island (*Neso1-8*, *10*–*13*, *15*–*23*), 14 in the *N. wilkinsi* and *N. questi* on Nightingale (*Neso1, 3, 4, 7, 9, 11, 13–15, 17, 18, 20, 22, 23*), and 7 in *R. goughensis* (*Neso5, 9, 13–15, 17, 23*). Bootstrap support <70% are not shown.

In total, 508 sequences of expected length (159 bp) were obtained from 14 *Nesospiza* from the Tristan da Cunha archipelago (10 from Inaccessible and 4 from Nightingale) and two *Rowettia goughensis* from Gough Island (see Figure 1). Only sequences that were found in two or more individuals were included (396 sequences), and among these, 23 unique alleles were identified (Figure 2; Additional file 1 Table S1). Since the MHC complex contains several paralogous loci, alleles cannot be assigned to a particular locus. This prevents the use of the standard nomenclature of MHC alleles [42], and therefore alleles were named *Neso01* – *Neso23*. No stop codons or frameshift mutations were present in any of these alleles, although one of the sequences (*Neso02*) contained an in-frame two codon insert, resulting in a 165 bp sequence. BLAST analysis indicated high similarity (87-96%, with coverage of 80-98%) of 21 alleles (*Neso01- Neso21*) to functional passerine MHCII alleles, whereas *Neso22* and *Neso23* had higher similarity (92-93%, with 98% coverage) to passerine pseudogenes.

Each individual *Nesospiza* contained 3–7 unique presumably functional (i.e. excluding known pseudogenes *Neso22* and *Neso23*) alleles of MHCIIβ (average ± SD: 4.63 ± 0.99). Assuming all loci to be heterozygous, the minimum number of MHCIIβ loci that must be present in *Nesospiza* is four. This is similar to what has been observed in most passerine species (3–7 loci), with the exception of common yellowthroat (*Geothlypis trichas*) (20 loci), which has particularly high levels of gene duplication [43]. A regression analysis performed to determine if the number of alleles sampled approached the maximum for each individual showed that the number of alleles did not plateau for 13 of the 16 individuals as the number of sequence clones increased (data not shown); thus, it is likely that more than four MHCIIβ loci are present in *Nesospiza*.

Of the 23 alleles, 21 were found in the *N. acunhae* individuals on Inaccessible Island (*Neso1-8*, *10*–*13*, *15*–*23*), 14 in the *N. wilkinsi* and *N. questi* on Nightingale (*Neso1, 3, 4, 7, 9, 11, 13–15, 17, 18, 20, 22, 23*), and 7 in *R. goughensis* (*Neso5, 9, 13–15, 17, 23*). The nucleotide diversity (π) of putatively functional alleles (i.e. excluding the pseodogenes, *Neso22* and *Neso23*) was 0.11 in *N. acunhae* on Inaccessible Island (data from 19 alleles in 10 individuals), 0.11 in *N. wilkinsi* on Nightingale (data from 8 alleles in 2 individuals), and 0.07 in *N. questi*on Nightingale (data from 7 alleles in 2 individuals). The nucleotide diversity (0.04) found in *R. goughensis* (data from the 6 alleles in 2 individuals)*.*

### Selection and recombination

The PBR was identified after alignment with the human HLA-DRB*04 amino acid sequence. Traditional selection statistics did not uncover any statistically significant selection patterns (Tajima’s D = 0.61, p > 0.10; Fu & Li’s D* = 0.30, p > 0.10; Fu & Li’s F* = 0.46, p > 0.10). The sampled populations showed no evidence of selection at the either the PBR or non-PBR regions (Table 1). Null models were supported by likelihood ratio tests, with only one site likely to be under positive selection (Table 2). Tests for recombination in RDP3 Beta 27 revealed no significant recombination events.

**Table 1:** **Proportion of non-synonymous (d**_**N**_**) and synonymous (d**_**S**_**) substitutions in MHCIIβ sequences of *Nesospiza *and other passerines**

		**PBR**				**Non-PBR**			
**Comparisons**	**N**	**d**_**N**_**(±SE)**	**d**_**S**_**(±SE)**	**d**_**N**_**/d**_**S**_	**z-test**	**d**_**N**_**(±SE)**	**d**_**S**_**(±SE)**	**d**_**N**_**/d**_**S**_	**z-test**
Brown *et al.*[44]									
*Neso01-23*	23	0.377 (±0.146)	0.236 (±0.161)	1.60	n.s. (0.54)	0.133 (±0.029)	0.114 (±0.051)	1.17	n.s. (0.74)
*Neso01-21 ǂ*	21	0.372 (±0.142)	0.234 (±0.174)	1.59	n.s. (0.59)	0.090 (±0.022)	0.097 (±0.047)	0.93	n.s. (0.90)
Group1	2	0.031 (±0.031)	0.000 (±0.000)	n/a	n.s. (0.33)	0.011 (±0.011)	0.039 (±0.040)	0.28	n.s. (0.51)
Group2	15	0.009 (±0.006)	0.000 (±0.000)	n/a	n.s. (0.16)	0.028 (±0.007)	0.042 (±0.030)	0.67	n.s. (0.68)
Group3	6	0.073 (±0.033)	0.036 (±0.049)	2.03	n.s. (0.51)	0.023 (±0.010)	0.014 (±0.014)	1.64	n.s. (0.62)
Inaccessible *ǂ*	19	0.361 (±0.133)	0.222 (±0.149)	1.63	n.s. (0.53)	0.135 (±0.026)	0.115 (±0.050)	1.17	n.s. (0.74)
Nightingale *ǂ*	12	0.420 (±0.151)	0.281 (±0.192)	1.49	n.s. (0.60)	0.166 (±0.034)	0.118 (±0.055)	1.41	n.s. (0.43)
Tristan da Cunha *ǂ*	21	0.377 (±0.146)	0.236 (±0.161)	1.60	n.s. (0.54)	0.133 (±0.029)	0.114 (±0.051)	1.17	n.s. (0.74)
*Rowettia goughensis*	6	0.486 (±0.188)	0.351 (±0.246)	2.59	n.s. (0.70)	0.194 (±0.040)	0.135 (±0.063)	1.44	n.s. (0.40)
Tong *et al.*[45]									
*Neso01-23*	23	0.230 (±0.090)	0.125 (±0.159)	1.84	n.s. (0.61)	0.174 (±0.033)	0.143 (±0.051)	1.22	n.s. (0.61)
*Neso01-21 ǂ*	21	0.100 (±0.054)	0.090 (±0.147)	0.11	n.s. (0.96)	0.151 (±0.032)	0.135 (±0.055)	1.12	n.s. (0.79)
Group1	2	0.053 (±0.052)	0.000 (±0.000)	n/a	n.s. (0.33)	0.053 (±0.053)	0.000 (±0.000)	n/a	n.s. (0.32)
Group2	15	0.018 (±0.019)	0.000 (±0.000)	n/a	n.s. (0.38)	0.024 (±0.006)	0.033 (±0.025)	0.73	n.s. (0.73)
Group3	6	0.033 (±0.022)	0.000 (±0.000)	n/a	n.s. (0.14)	0.036 (±0.013)	0.024 (±0.017)	1.50	n.s. (0.58)
Inaccessible *ǂ*	19	0.240 (±0.091)	0.126 (±0.165)	1.90	n.s. (0.57)	0.171 (±0.032)	0.139 (±0.049)	1.13	n.s. (0.59)
Nightingale *ǂ*	12	0.306 (±0.124)	0.152 (±0.189)	2.01	n.s. (0.54)	0.204 (±0.038)	0.152 (±0.058)	1.34	n.s. (0.44)
Tristan da Cunha *ǂ*	21	0.230 (±0.090)	0.125 (±0.159)	1.84	n.s. (0.61)	0.174 (±0.033)	0.143 (±0.051)	1.22	n.s. (0.61)
*Rowettia goughensis*	6	0.334 (±0.123)	0.163 (±0.189)	2.05	n.s. (0.52)	0.238 (±0.045)	0.186 (±0.069)	1.28	n.s. (0.49)
New Zealand robin	41	0.339 (±0.078)	0.094 (±0.059)	3.6	<0.005	0.076 (±0.019)	0.039 (±0.013)	1.95	n.s.
Chatham Island robin	4	0.373 (±0.086)	0.135 (±0.078)	2.76	<0.05	0.099 (±0.024)	0.020 (±0.014)	5.05	<0.005
Hawaiian honeycreepers	51	0.341 (±0.103)	0.076 (±0.095)	4.49	<0.001	0.121 (±0.038)	0.092 (±0.053)	1.32	n.s.
Common yellowthroat	39	0.608 (±0.120)	0.211 (±0.111)	2.88	<0.05	0.135 (±0.034)	0.137 (±0.034)	0.99	n.s.
House sparrow*	12	0.470 (±0.109)	0.123 (±0.095)	3.82	<0.0001	0.203 (±0.047)	0.200 (±0.051)	1.02	n.s.

**References: New Zealand and Chatham Island robins**[34,35], **Hawaiian honeycreepers**[46], **common yellowthroat**[43];* Values were calculated from GenBank sequences, ^ǂ^ Does not include the putative pseudogenes *Neso22* and *Neso23*, N = Number of MHC sequences; Brown *et al.*[44]**: PBR = 14 amino acids, non-PBR = 41 amino acids; Tong *et al.***[45]**: PBR = 9 amino acids, non = PBR = 46 amino acids.**

**Table 2:** Parameter estimates and results from four selection models as implemented in CODEML

**Model**	**Log-likelihood**	**Parameter estimates**	**Positively selected sites**
M1a (nearly neutral)	−576.502	p_0_ = 0.385, p1 = 0.615, ω0 = 0.038,	Not allowed
		ω1 = 1.000	
M2a (positive selection)	−574.685	p_0_ = 0.311, p1 = 0.628, p2 = 0.061 ,	None
		ω0 = 0.000, ω1 = 1.000 , ω2 = 4.847	
M7 (beta)	−576.474	p = 0.033, q = 0.018	Not allowed
M8 (beta and omega)	−574.720	p_0_ = 0.941, p1 = 0.059, p = 0.028,	37 N
		q = 0.015, ω = 4.612	

### Phylogenetic analysis

**Figure 3:**
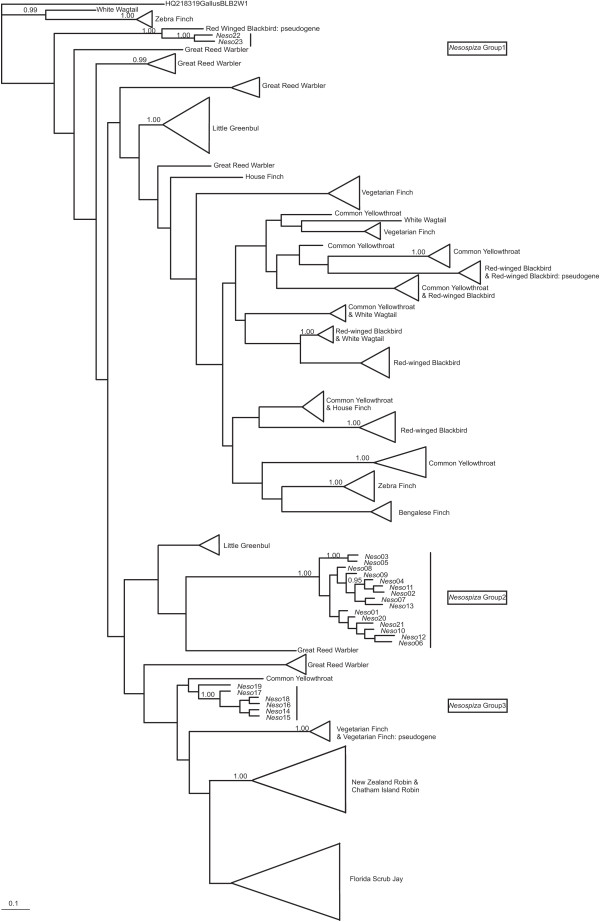
**Passerine MHCIIβ gene tree.** Gene tree of the MHCIIβ exon 2 sequences of *Nesospiza* and sequences of several other passerine species obtained from GenBank. A Bayesian analysis of 159 bp of sequences, with independent mutational models applied to each codon position (Position 1: TIM3ef + I + G; Position 2: TVM + G; Position 3: TPM2uf + G). Bayesian posterior probabilities are indicated at the nodes; values < 0.95 are not shown.

A consensus Neighbour-Joining tree of the 23 *Nesospiza* alleles showed three highly supported groups, called *Nesospiza* Group 1 – 3 (Figure 2). The same three *Nesospiza* groups were highly supported within genealogies for passerine MHCIIβ reconstructed from exon 2 sequences using Bayesian inference (Figure 3). Group 1, containing the *Neso22* and *Neso23*, and a red-winged blackbird pseudogene (*Agelaius phoeniceus*; APAF030990), form a highly supported, diverged cluster. A second red-winged blackbird pseudogene (APAF030994) and a vegetarian finch (*Platyspiza crassirostris*) pseudogene (PCAY064469), however, group with other presumably functional passerine MHC sequences.

Group 2 (*Neso01-13, 20–21*) is distinct and appears to be a well-supported cluster of presumably functional MHC alleles unique to *Nesospiza* and *R. goughensis*. Group 3 (*Neso14-19*), which also contains sequences shared by *Nesospiza* and *R. goughensis,* is well supported, but clusters more closely with sequences from the distantly related common yellowthroat, New Zealand robin (*Petroica australis*), Chatham Island robin (*Petroica traverse*), Florida scrub jay (*Aphelocoma coerulescens*) and vegetarian finch. Of the other passerine species, zebra finch, Florida scrub jay, and little greenbul (*Andropadus virens;* with the exception of one sample) cluster by species or, in the case of New Zealand and Chatham Island robins (Petroica australis), with sister species. Sequences of the great reed warbler (*Acrocephalus arundinaceus*) are scattered throughout the phylogeny as small groups or single alleles, apart from one supported group divergent from most other passerine sequences. The sequences of several passerines, namely house finch (*Carpodacus mexicanus*), vegetarian finch, red-winged blackbird, and common yellowthroat, cluster with those of other species throughout the phylogeny.

## Discussion

This study describes 23 MHCIIß alleles representing at least four functional loci and two pseudogenes in the *Nesospiza* bunting species complex. Many MHCIIβ alleles were shared between *Nesospiza* taxa as well as between *Nesospiza* and its putative sister taxon *R. goughensis.* This pattern of ancestral polymorphism suggests that the observed gene duplications occurred prior to the phylogenetic split of the species, and subsequent unusually low selective pressure at the loci has prevented allelic divergence between species. The MHC nuclear genetic diversity in *Nesospiza* on Inaccessible (π = 0.11) was comparable to that of outbred passerine species (e.g. 0.15 in *Luscinia svecica*; [5]), and despite the low sample size for Nightingale, allele numbers and nucleotide diversity were higher than in the severely bottlenecked Chatham Island robin population (0.05) [35]. We have screened 14 *Nesospiza* individuals for MHC variation, which is similar to some previous Passerine MHC studies using cloning and sequencing e.g. [34,35,43,47]. However, because larger sample sizes would have been necessary to cover the variation of each population sufficiently, we will not discuss population-level MHC variation further.

Patterns of both ancestral polymorphism and concerted evolution among *Nesospiza* and *Rowettia* populations are evident from our results. Ancestral polymorphism, found here for *Nesospiza* and *R. goughensis*, as well as in other species (e.g. great reed warbler, house finch, vegetarian finch, red-winged blackbird and common yellowthroat), can be seen in the sharing of the same or similar alleles between species (Figures 2 and 3). Of the 23 *Nesospiza* alleles, 15 were found in species from both islands. All seven alleles occurring in *R. goughensis* are shared with *Nesospiza* (*Neso5, 9, 13–15, 17, 23*) and these alleles are found in all three *Nesospiza* groups in the gene tree (Figures 2 and 3). The estimated minimum number of putatively functional gene copies in *Nesospiza* (i.e. 4 loci) suggests that the three *Nesospiza* allele groups are not necessarily locus-specific, despite their divergent clustering. Group 3 may represent a single locus, since only one or two alleles from this cluster occur in each individual. However, this is not the case for *R. goughensis*, where three of these alleles occur in one individual. Two highly supported clusters are seen within Group 2 (Figure 3), which is also the cluster containing the most alleles, suggesting that this cluster is likely to represent more than one gene copy. A likely explanation for the clustering of alleles from different gene loci is the genetic homogenization caused by gene duplication events with subsequent gene conversion.

The highly supported branches of sequences forming Groups 2 and 3 in the gene tree contain only *Nesospiza* and *R. goughensis* alleles. Although several species were included due to the similarity between their MHCIIβ alleles and those of *Nesospiza*, the observed divergent clustering of Group 2sequences could be explained by a lack of closely related species in the analysis. Alternatively, the species-specific clustering of *Nesospiza* may be attributed to their long divergence time from the other passerines sampled [48]. The deep divergence of Groups 2 and 3, and the clustering of Group 3 close to the distantly related species of common yellowthroat, New Zealand robins, Florida scrub jay, and vegetarian finch, however, provide strong support for preserved ancestral polymorphism. These patterns suggest that extant MHC variation in *Nesospiza* and *R. goughensis* can be explained by shared ancestral polymorphism during colonisation which has since been maintained. It is possible that the additional variation has been generated by gene conversion events, which is the most likely method of generating variation from the few alleles remaining in a population following a population bottleneck [26].

**Figure 4:**
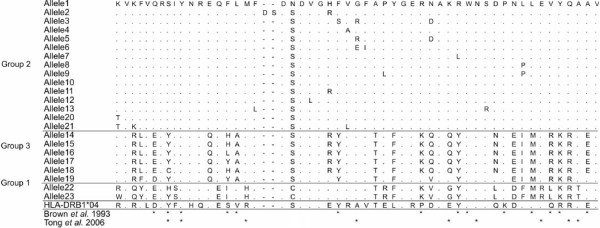
**Assignment of *Nesospiza *MHCIIβ peptide binding sites.** Alignment of MHCIIβ exon 2 amino acid sequences of *Neso01 – 23* indicating amino acid differences between Groups 1–3 sequences. Amino acid identity is shown by “.” and an alignment gap by “-”. Alignment with human HLA-DRB*04 (GenBank accession: NM_021983) was used to assign peptide binding sites (*) according to Brown *et al.*[44] and Tong *et al.*[45]

Amino acid sequences are more similar between Groups 1 and 3 (Figure 4). This could either represent evidence of recombination with the pseudogenes, producing a new group of functional sequences, or perhaps more likely indicate that the pseudogenes resulted from gene duplication events of Group 3 sequences. Copying errors during gene duplication and recombination events may result in non-functional genes (pseudogenes) and the subsequent lack of functional constraint on evolutionary processes (such as mutation) acting on the pseudogenes result in rapid sequence divergence [49]. This is evidently the case for the two presumably non-functional alleles, *Neso22* and *Neso23*, which form a well supported group with a red-winged blackbird pseudogene, clustered sister to all the functional passerine sequences. However, some pseudogenes (e.g. red-winged blackbird APAF030994 and vegetarian finch PCAY064439) do not show evidence of rapid divergence (Figure 3), perhaps due to ongoing recombination with functional genes that is leading to sequence conservation. Alternatively, there may have been insufficient time for the genes to become highly diverged since they became non-functional.

Selection tests showed no consistent evidences of balancing or positive selection at the PBR or non-PBR regions of MHCIIβ exon 2 in *Nesospiza* and *Rowettia*. The short fragment length of our sequences excludes some of the PBR sites, and therefore there is a chance that some sites that may be under selection were excluded from the analyses. However, selection tests were done according to two different PBR characterizations [44,45], and tested on the entire data set as well as all species individually, and the three clusters independently. Ratios of d_N_/d_S_ were non-significant in all cases (Table 1), and additional selection tests showed weak evidence of selection with only one site likely to be under positive selection (Table 2). New MHC variation can be generated by point mutations or through recombination between alleles, giving rise to a new allele [26,33]. The latter process, known as gene conversion, has been documented in some natural avian populations [22,25,26] and has been suggested to be essential in generating genetic variation at MHC after a bottleneck [26]. During gene conversion events, synonymous substitutions may hitchhike with non-synonymous variation [26] and this may be a reason why d_N_/d_S_ ration tests fail to detect positive selection. We found, however, no evidence of recombination in our data, but recombination can be difficult to verify with short sequences.

Despite the lack of significant evidence for selection, ratios of d_N_/d_S_ > 1.0 that we observe in *Rowettia* and all *Nesospiza* populations indicate that the loci are under weak balancing selection, or perhaps more likely, that ancestral balancing selection acted on the loci before colonisation of the islands. Lack of strong positive selection may reflect a decreased pathogen load in both *Nesospiza* and *R. goughensis*. Passerines generally are less parasitised by lice and ectoparasites than other avian orders e.g. [50]. This is particularly true of small populations on isolated oceanic islands (R Palma pers. comm.). *Myrsidea* lice occur at extremely low prevalence (6.4%) across 12 species of Darwin’s finches at the Galápagos Islands [50]. On Tristan da Cunha and Gough Island, different louse species (order Phthiraptera) have been found on 20 bird species, including the Tristan thrush (*Nesocichla eremita*) [51], yet careful inspection of *Nesospiza* buntings yielded no lice, with hippoboscid flies and feather mites the only ectoparasites (PG Ryan unpubl. data). The absence of parasites could be due to an uninfected founding population (“missing the boat”) [52], or subsequent extinction from the host after colonisation. The high level of ancestral polymorphism between *R. goughensis* and *Nesospiza* suggest that the former is more likely, where a single uninfected founding population colonization both Tristan da Cunha and Gough Island.

Some shortcomings of the cloning and sequencing method employed in the study may result in underestimation of MHC variation. Firstly, the large number of gene copies and the high level of convergence between loci make it difficult to amplify a single MHC locus at a time. Thus, most MHC studies on non-model vertebrates amplify alleles from multiple gene copies simultaneously. This increases the risk of chimera formation during the PCR, which in turn leads to overestimation of levels of gene recombination [53]. In addition, PCR products are prone to point mutations e.g. [54], although these are relatively easy to detect since mutation rates are relatively low and are unlikely to occur in more than one sequence [55,56]. In this study, we compensate for these problems by only accepting alleles that occur in at least two individuals e.g. [57,58]. Secondly, the amplification of a multi-gene family is necessarily problematic since not all loci and not all alleles at a locus will be detected using a single primers set. The primers employed in this study were designed for non locus-specific amplification of exon 2 of MHCIIß in zebra finch (*Taeniopygia guttata*) [59] and have been successfully employed in other passerine MHC studies (H Westerdahl pers. comm.). A regression analysis of the number of clones sequenced per individual found that more individuals and sequences will be necessary to estimate true MHC variation per individual. Finally, sequences were obtained for only half of the variable MHCIIβ exon 2 gene. Although not all the variation has been analysed in this study, this is often the case with such complex multi-gene systems [58] and does not preclude our finding of ancestral polymorphism between species and within the *Nesospiza* species complex. More comprehensive studies of population level variation of MHC would require that more individuals and sequences were analysed. However, the present study focuses on selection and levels of shared polymorphism, and for such analyses the present data is sufficient.

## Conclusions

The extent of shared alleles and ancestral polymorphism between *Nesospiza* and *R. goughensis* suggests that both originated from the same colonization wave. We find that similar or the same alleles are maintained between species due to the recent species divergence and low levels of (local) selection acting on PBR. The additional variation found within the *Nesospiza* species complex may be due to gene conversion, which is likely the most prominent mechanism for generating new variation after a bottleneck event [26]. The extant genetic variation is not likely to change rapidly, unless there is a drastic geographic or environmental change leading to strong selection at the MHC. One such situation would be the introduction of pathogens, since populations with low MHC diversity are often more susceptible to novel pathogens [35,60]. In the absence of strong selection, MHC is expected to diverge over time between islands and populations due to drift, with the generation of new haplotypes through point mutations or gene conversion. Ongoing gene flow between populations and subspecies on Inaccessible Island can maintain genetic variation to some extent. The potential role of MHC dependent sexual selection [22,61] to drive divergence between populations even further remains open to study, and would require wider sampling over the entire geographic range to cover the details of geographic- and species-specific variation.

## Methods

### Sampling

Buntings were mist-netted or caught with hand nets at Inaccessible, Nightingale and Gough Islands during September 1999 – February 2000, with additional samples from Inaccessible Island collected in September – November 2004 [37,38]. No extant *Nesospiza* species occur on Tristan Island. Brachial vein blood samples were collected and stored in EDTA or lysis buffer. Two to three individuals were chosen to represent each population (Figure 1; Inaccessible: 3 *N. a. acunhae*, 2 *N. a. fraseri*, 2 *N. a. dunnei*, 3 *N. a*. hybrid; Nightingale: 2 *N. questi*, 2 *N. wilkinsi*; Gough Island: 2 *R. goughensis*).

### DNA extraction and amplification

DNA was extracted from whole blood by standard phenol:chloroform methods [Sambrook]. The primers 2zffw1 (5’ TGT CAC TTC AYK AAC GGC ACG GAG 3’) and 2zfrv1 (5’ GTA GTG TGC CGG CAG TAC GTG TC 3’), previously designed for the zebra finch (*Taeniopygia guttata*) [59], were used to amplify 159 bp of MHCIIß exon 2. These primers are not locus-specific and amplify exon 2 of multiple copies of the MHCIIß gene. Amplifications were performed in 10 μl volumes, each containing 5 μl QIAGEN Multiplex PCR Master Mix, 10 pM of each primer, and 10 ng of template DNA. PCR cycling conditions involved an initial denaturing step of 15 minutes at 95C, followed by 35 cycles of 30 seconds at 94C, 1 minute 30 seconds at 64C and 1 minute 30 seconds at 72C.

### Cloning and sequencing

PCR products of all individuals were cloned using the TOPO TA Cloning^®^ kit (Invitrogen). Vectors (pCR^®^ 2.1-TOPO^®^) with inserted PCR product were used to transform chemically competent *Escherichia coli* cells (OneShot^®^), according to the manufacturer’s instructions. Transformed cells were cultured on S.O.C medium (Invitrogen) for one hour in a shaking incubator at 37C and then incubated overnight at 37C on LB-medium supplemented with 50 μg/ml Ampicillin and 50 μl of X-gal (40 mg/ml). For each sample 30 positive colonies were picked with a sterile toothpick, diluted in 100 μl Sabax water (Adcock Ingram) and used directly as DNA template for PCR. Amplification reactions contained 2 μl QIAGEN Multiplex Master Mix, 10 pM each of M13 forward and M13 reverse primers (included in the kit), and 2 μl of the colony diluted in Sabax water. The same PCR cycling conditions were used as before (see above). All clones were sequenced in both directions on an ABI Prism 3100 capillary sequencer (Applied Biosystems). A total of 12 – 29 clones were successfully sequenced per individual (average = 22.88).

### Data analysis

Nucleotide sequences were edited and aligned using CLC Main Workbench 5.0.2 (CLC Bio). To avoid including false haplotypes due to artefacts arising during PCR (e.g. recombinant chimeric sequences), sequences were only accepted if they were present in two or more individuals [56,62] (396 of 508 sequences were accepted and these represented 23 different alleles; Additional file 1 Table S1). Due to the large number of sequences excluded with this stringent method, we followed Anmarkrud *et al.*[5] suggestion to identify additional true alleles and evaluated whether the excluded sequences were >1.5% (~3 bp) different from any of the sequences that were identified as possible alleles. Only two of the excluded sequences differed with >1.5% and since so few alleles would not affect the results we decided not to include them in the analyses.

The nucleotide diversity (π) was calculated using DnaSP 5.0 [63]. Sequences were verified as MHC alleles using the BLASTN 2.2.24 algorithm [64] available through the National Center for Biotechnology Information (NCBI). Of the 23 alleles identified, 21 (*Neso01 – 21*) showed high identity (87-96%, with coverage of 80-98%) to known passerine MHCIIß coding genes, and two alleles (*Neso22* and *Neso23*) showed high identity (92-93%, with 98% coverage) with passerine pseudogenes (Figure 3). This suggests that *Neso22* and *Neso23* are non-functional, thus they were excluded from the selection tests.

A regression analysis was performed to determine if the number of sequences obtained for each individual effectively sampled the total number of alleles. For each individual, a random subset of the alleles obtained was sampled and the number of alleles in the subset counted. This was repeated 100 times each for a subset of 5, 10, 15, 20 and 25 (restricted by the number of sequences obtained for each individual). As sampling approaches the maximum number of alleles in the population, the number of alleles found in increasing subset sizes will plateau.

Nucleotide positions associated with the PBR were assigned according to the PBR regions determined for the human antigen binding region by two different studies [44,45]. Selection was tested using the ratio of nonsynonymous (d_N_) to synonymous (d_S_) substitutions (d_N_/d_S_ = ω). Under strict neutrality d_N_ = d_S_, while regions under balancing selection are expected to undergo more nonsynonymous substitutions and regions under directional selection more synonymous substitutions. The parameter ω was calculated in MEGA 4 [65] using the method of Nei and Gojobori [66] with Jukes Cantor corrections and 1000 bootstrap replicates. A z-test [66] was used to determine the probability of selection by comparing the selection parameter, ω, against a null hypothesis of strict neutrality (d_N_ = d_S_). Standard selection tests (Tajima’s D, Fu & Li’s F* and Fu & Li’s D*) were calculated in DnaSP 5 [63]. Substitution rates, ω, and the probability of positive selection on PBR and non-PBR regions, were compared to results from New Zealand and Chatham Island robins (*Petroica australis* and *Petroica traverse*) [34,35], Hawaiian honeycreepers (*Drepanidinae*) [46], common yellowthroat (*Geothlypis trichas*) [43], and house sparrow (*Passer domesticus*; values calculated using sequences from GenBank).

In a second test of selection, the maximum likelihood method implemented in CODEML in the Phylogenetic Analysis by Maximum Likelihood package (PAML 3.14) [67,68], was used to identify the sites under selection. Likelihood ratio tests in CODEML were used to test neutral models and models of selection. In a first comparison, a neutral model M1a (ω_0_ < 1, ω_1_ = 1) was tested against M2a, a model for positive selection (ω_2_ > 1). Model M1a assumes that sites are either conserved or under purifying selection (i.e. removed from the population) (ω_0_ < 1), or selectively neutral (ω_1_ = 1). Model M2a considers a third class of sites where sites may be under positive selection (ω_2_ > 1). A second comparison tested a neutral model M7 (0 < ω < 1) against a model for positive selection, M8 (0 < ω < 1, ω > 1). Model M7 is based on a β distribution and estimates ω as a value between 0 and 1. In M8, ω is estimated directly from the data for one class of sites which allows for ω > 1. Both these tests are used routinely to identify sites under selection [69]. The best-fit model was determined using a likelihood ratio test for each model comparison, thus the likelihood of positive selection could be evaluated [70]. The difference in likelihood values of the null model (M1a, M7) and the alternative model (M2a, M8) was compared with the χ^2^ distribution. Degrees of freedom were calculated as the difference in the number of parameters for each test. The Bayes Empirical Bayes method, implemented in CODEML, was used to calculate the posterior probability for each site class for the M2a and M8 models. A site is likely to be under positive selection when the posterior mean of ω > 1 [68].

To determine the phylogenetic relationship between the 23 *Nesospiza* alleles a Neighbour-Joining (NJ) tree was constructed in MEGA 4 [65] assuming homogenous substitution patterns among lineages and uniform rates among sites. A consensus tree was computed from 10 000 bootstrap replicates in MEGA 4 [65] using a 75% consensus cut-off value. All subsequent phylogenetic analyses were conducted in MrBayes v 3.1.2 [70]. A concatenated data set comprising MHCIIβ sequences from several passerines obtained from GenBank (Figure 3) was analysed with all *Nesospiza* alleles (*Neso01* – *Neso23*). The passerine species most closely related to *Nesospiza*, chosen as the top ten hits for each *Nesospiza* allele using BLAST, and several other passerine species (chosen to represent passerine diversity), were used for the phylogenetic analyses. Sequences were only included if there was sequence alignment of more than 100 bp, thus some species (e.g. *Poephila acuticauda*) identified to be in the top ten closest matches to one of the *Nesospiza* alleles were not included. This cut-off was made to ensure a robust result from the phylogenetic analysis.

The best model for nucleotide substitution was chosen using the Akaike Information Criterion (AIC) [71] as determined by jModelTest [72,73] for each codon position independently (Position 1: TIM3ef + I + G; Position 2: TVM + G; Position 3: TPM2uf + G). Divergent zebra finch sequences were chosen as a root for passerine MHCIIβ [60]. MrBayes was run for 3 million generations with four incrementally heated chains. Trees were sampled every 3 000 generations, with a 10% burn-in. A consensus tree and posterior probabilities were calculated from the sampled trees. The average standard deviation of split frequencies between two simultaneous runs was monitored to confirm convergence.

The RDP3 Beta 27 [74] package was used to test for signatures of recombination using multiple algorithms simultaneously: RDP [75], GENECONV [76], BootScan [77], MaxChi [78], Chimaera [79], and 3Seq [80]. The default settings were used, and the significance level was set to 0.05. Bonferroni corrections were applied for multiple comparisons [81].

### Additional material

GenBank accession numbers of non-*Nesospiza* sequences used in the present study: L42334 - L42335, U23968 - U23969, U23967, U23970, U23971, AJ404371 - AJ404376, U24405, AY437900 - AY437912, AY428561 - AY428568, AY258333 - AY248335, AY428569, U23958 - U23966, U23972, U23973, U23975, XM_002192161, XM_002193356, XM_002196138, XM_002197722, XM_002198130, XM_002198161, XM_002199709, XM_ 002200257, AF165156 - AF165157, AF165159, Z74424 - Z74428, AY064425, AY064439, AY064451, GQ247601 - GQ247606, GQ247608 - GQ247609, GQ247613 - GQ247614, GQ247616 - GQ247622, GU390288 - GU390291, AY518171 - AY518183, AY583092 - AY583094.

## Authors’ contributions

AJvR carried out the molecular lab work, statistical analyses, and drafted the manuscript. BH, PB, and PGR conceived of the study and participated in its design. BH participated in the coordination of the study and helped to draft the manuscript. All authors read and approved the final manuscript.

## Supplementary Material

Additional file 1 **Table S1. **List of 23 major histocompatibility complex class II ß (MHCIIß) exon 2 *Nesospiza* bunting sequences used in the present study. Click here for file

## References

[B1] Dlugosch KM Parker: Founding events in species invasions: genetic variation, adaptive evolution, and the role of multiple introductions MolEcol 2008 17 431 449 10.1111/j.1365-294X.2007.03538.x 17908213

[B2] Lande R Shannon S The role of genetic variation in adaptation and population persistence in a changing environment Evolution 1996 50 434 437 10.2307/2410812 28568879

[B3] Robertson A Selection for heterozygotes in small populations Genetics 1962 47 1291 1300 13974344 10.1093/genetics/47.9.1291 PMC1210407

[B4] Oliver MK Piertney SB Selection maintains MHC diversity through a natural population bottleneck MolBiolE 2012 in press 10.1093/molbev/mss063 22323362

[B5] Anmarkrud JA Johnsen A Bachmann L Lifjeld T Ancestral polymorphism in exon 2 of bluethroat (*Lusciniasvecica*) MHC class II B genes J EvolBiol 2010 23 1206 1217 10.1111/j.1420-9101.2010.01999.x 20456568

[B6] Hess CM Edwards SV The evolution of the major histocompatibility complex in birds Bioscience 2002 52 423 431 10.1641/0006-3568(2002)052[0423:TEOTMH]2.0.CO;2

[B7] Ekblom R Sæther SA Jacobsson PAR Fiske P Sahlman T Grahn M Kålås JA Höglund J Spatial pattern of MHC class II variation in the great snipe (*Gallinago media*) MolEcol 2007 16 1439 1451 10.1111/j.1365-294X.2007.03281.x 17391268

[B8] Klein J Natural history of the major histocompatibility complex 1986 New York: John Wiley & Sons

[B9] Parham P Ohta T Population biology of antigen presentation by MHC class I molecules Science 1996 272 67 74 10.1126/science.272.5258.67 8600539

[B10] Gaudieri S Dawkins RL Habara K Kulski JK Gojobori T SNP profile within the human major histocompatibility complex reveals an extreme interrupted level of nucleotide diversity Genome Res 2000 10 1579 1586 10.1101/gr.127200 11042155 PMC310975

[B11] Doherty PC Zinkernagel RM Enhanced immunological surveillance in mice heterozygous at the H-2 complex Nature 1975 256 50 52 10.1038/256050a0 1079575

[B12] Penn DJ Potts WK The evolution of mating preferences and major histocompatibility complex genes Am Nat 1999 153 145 164 10.1086/303166 29578757

[B13] Spurgin LG Richardson DS How pathogens drive genetic diversity: MHC, mechanisms and misunderstandings Proc R Soc Lond B Biol Sci 2010 277 979 988 10.1098/rspb.2009.2084 PMC2842774 20071384

[B14] Takahata N Satta Y Klein J Polymorphism and balancing selection at major histocompatibility loci Genetics 1992 130 925 938 1582567 10.1093/genetics/130.4.925 PMC1204941

[B15] Oliver MK Telfer S Piertney SB Major histocompatibility complex (MHC) heterozygote superiority to natural multi-parasite infections in the water vole (*Arvicolaterrestris*) Proc R Soc Lond B Biol Sci 2009 276 1119 1128 10.1098/rspb.2008.1525 PMC2679068 19129114

[B16] Penn DJ The scent of genetic compatibility: sexual selection and the major histocompatibility complex Ethology 2002 108 1 21 10.1046/j.1439-0310.2002.00768.x

[B17] Richardson DS Komdeur J Burke T von Schantz T MHC-based patterns of social and extra-pair mate choice in the Seychelles warbler Proc R Soc B 2005 272 759 767 10.1098/rspb.2004.3028 PMC1602051 15870038

[B18] van Oosterhout C A new theory of MHC evolution: beyond selection on the immune genes Proc R Soc B 2009 276 657 665 10.1098/rspb.2008.1299 PMC2660941 18986972

[B19] Nei M Rooney AP Concerted and birth-and-death evolution of multigene families Annu Rev Genet 2005 39 121 152 10.1146/annurev.genet.39.073003.112240 16285855 PMC1464479

[B20] Edwards SV Grahn M Potts WK Dynamics of MHC evolution in birds and crocodilians: amplification of class II genes with degenerate primers MolEcol 1995 4 719 729 10.1111/j.1365-294x.1995.tb00272.x 8564010

[B21] Westerdahl H Wittzell von Schantz H Mhc diversity in two passerine birds: no evidence for a minimal essential Mhc Immunogenetics 2000 52 92 100 10.1007/s002510000256 11132162

[B22] Promerová M Albrecht T Bryja J Extremely high MHC class I variation in a population of a long-distance migrant, the Scarlet Rosefinch (*Carpodacuserythrinus*) Immunogenetics 2009 61 451 461 10.1007/s00251-009-0375-x 19452149

[B23] Nei M Gu X Sitnikova T Evolution by the birth-and-death process in multigene families of the vertebrate immune system PNAS 1997 94 7799 7806 10.1073/pnas.94.15.7799 9223266 PMC33709

[B24] Gu X Nei M Locus specificity of polymorphic alleles and evolution by a birth-and-death process in mammalian MHC genes MolBiolEvol 1999 16 147 156 10.1093/oxfordjournals.molbev.a026097 10028282

[B25] Witzell H Bernot A Auffrey C Zoorob R Concerted evolution of two MHC class II B loci in pheasants and domestic chickens MolBiolEvol 1999 16 479 490 10.1093/oxfordjournals.molbev.a026130 10331274

[B26] Spurgin LG van Oosterhout C Illera JC Bridgett S Gharbi K Emerson BC Richardson DS Gene conversion rapidly generates histocompatibility complex diversity in recently founded bird populations MolEcol 2011 20 5213 5225 10.1111/j.1365-294X.2011.05367.x 22106868

[B27] Takahata N Nei M Allelic genealogy under overdominant and frequency-dependent selection and polymorphism of Major Histocompatibility Complex loci Genetics 1990 124 967 978 2323559 10.1093/genetics/124.4.967 PMC1203987

[B28] Klein J Origin of Major Histocompatibility Complex polymorphism – the transspecies hypothesis Hum Immunol 1987 19 155 162 10.1016/0198-8859(87)90066-8 3305436

[B29] Figueroa F Gunther E Klein J MHC polymorphism in the MHC class II of a non-passerine bird, the great snipe (*Gallinago media*) Nature 1988 335 265 267 10.1038/335265a0 3137477

[B30] Lawlor DA Ward FE Ennis PD Jackson AP Parham P HLA-A and HLA-B polymorphism predate the divergence of human and chimpanzees Nature 1988 335 268 271 10.1038/335268a0 3412487

[B31] Bernatchez L Landry C MHC studies in nonmodel vertebrates: what have we learned about natural selection in 15 years? J Evolution Biol 2003 16 363 377 10.1046/j.1420-9101.2003.00531.x 14635837

[B32] Richardson DS Westerdahl H MHC diversity in two Acrocephalus species: the outbred great reed warbler and the inbred Seychelles warbler MolEcol 2003 12 3523 3529 10.1046/j.1365-294x.2003.02005.x 14629367

[B33] Bahr A Wilson AB The evolution of MHC diversity: Evidence of intralocus gene conversion and recombination in a single-locus system Gene 2012 497 52 57 10.1016/j.gene.2012.01.017 22301266

[B34] Miller HC Lambert DM Gene duplication and gene conversion in class II MHC genes of New Zealand robins (Petroicidae) Immunogenetics 2004 56 178 191 15138734 10.1007/s00251-004-0666-1

[B35] Miller HC Lambert DM Genetic drift outweighs balancing selection in shaping post-bottleneck major histocompatibility complex variation in New Zealand robins (Petroicidae) MolEcol 2004 13 3709 3721 10.1111/j.1365-294X.2004.02368.x 15548285

[B36] Rand AL The origin of landbirds of Tristan da Cunha, Nightingale and Inaccessible Islands Fieldiana Zoology 1955 37 139 166

[B37] Ryan PG Rensburg A Moloney S Grant TJ Delport W Ecological speciation in South Atlantic island finches Science 2007 315 1420 1423 10.1126/science.1138829 17347442

[B38] van Rensburg J Resolving the fine-scale population structure of Nesospizabuntings using a genetic multi-marker system 2011 University of Pretoria: MSc thesis

[B39] Ryan PG Taxonomic and conservation implications of ecological speciation in Nesospizabuntings on Tristan da Cunha Bird ConservInt 2008 18 20 29

[B40] Bollmer JL Vargas FH Parker PG Low MHC variation in the endangered Galápagos penguin (*Spheniscusmendiculus*) Immunogenetics 2007 59 593 602 10.1007/s00251-007-0221-y 17457582

[B41] Travis EK Vargas FH Merkel J Gottdenker N Miller RE Parker PG Hematology, serum chemistry, and serology of Galápagos penguins in the Galápagos Islands, Ecuador J Wildlife Dis 2006 42 625 632 10.7589/0090-3558-42.3.625 17092893

[B42] Klein J Bontrop RE Dawkins RL Erlich HA Gyllensten UB Heise ER Jones PP Parham P Wakeland EK Watkins DI Nomenclature for the major histocompatibility complexes of different species: a proposal Immunogenetics 1990 31 217 219 2329006 10.1007/BF00204890

[B43] Bollmer JL Dunn PO Whittingham LA Wimpee C Extensive MHC Class II B gene duplication in a passerine, the common yellowthroat (*Geothlypistrichas*) J Hered 2010 101 448 460 10.1093/jhered/esq018 20200139

[B44] Brown JH Jardetzky TS Gorga JC Stern LJ Urban RG Storminger JL Wiley DC Three-dimensional structure of the human class II histocompatibility antigen HLA-DR1 Nature 1993 364 33 39 10.1038/364033a0 8316295

[B45] Tong JC Zhang GL Tan TW August JT Brusic V Ranganathan S Prediction of HLA-DQ3.2β Ligands: evidence of multiple registers in class II binding peptides Bioinformatics 2006 22 1232 1238 10.1093/bioinformatics/btl071 16510499

[B46] Jarvi SI Tarr CL McIntosh CE Atkinson CT Fleischer RC Natural selection of the major histocompatibility complex (*MHC*) in Hawaiian honeycreepers (*Drepanidinae*) Mol Ecol 2004 13 2157 2168 10.1111/j.1365-294X.2004.02228.x 15245391

[B47] Bonneaud C Sorci G Morin V Westerdahl H Zoorob R Wittzell H Diversity of MHC class I and II B genes in house sparrows (*Passer domesticus*) Immunogenetics 2004 55 855 865 10.1007/s00251-004-0648-3 14963619

[B48] Aguilar A Edwards SV Smith TB Wayne RK Patterns of variation in MHC class II β loci of the little greenbul (*Andropadusvirens*) with comments on MHC evolution in birds J Hered 2006 97 133 142 10.1093/jhered/esj013 16489149

[B49] Swanson WJ Vacquier VD The rapid evolution of reproductive proteins Nat Rev Genet 2002 3 137 144 11836507 10.1038/nrg733

[B50] Palma RL Price RD The species of MyrsideaWaterston (Insecta: Phthiraptera: Menoponidae) from the Galápagos Islands, with descriptions of new taxa Tuhinga 2010 21 135 146

[B51] Hänel C Palma RL The lice of the Tristan da Cunha archipelago (Insecta: Phthiraptera) BeiträgeEntomol 2007 57 105 133

[B52] Paterson AM Gray RD Clayton DH, Moore J From Host-parasite co speciation, host switching and missing the boat Host-parasiteevolution: General principles and avian models 1997 Oxforda: Oxford University Press 236 250

[B53] Kanagawa T Bias and artifacts in multitemplate polymerase chain reactions (PCR) J Biosci Bioeng 2003 96 317 323 16233530 10.1016/S1389-1723(03)90130-7

[B54] Cline J Braman JC Hogrefe HH PCR fidelity of Pfu DNA polymerase and other thermostable DNA polymerases Nucleic Acid Research 1996 24 3546 3551 10.1093/nar/24.18.3546 PMC146123 8836181

[B55] Galan M Guivier E Caraux G Charbonnel N Cosson JF A 454 multiplex sequencing method for rapid and reliable genotyping of highly polymorphic genes in large-scale studies BMC Genomics 2010 11 296 10.1186/1471-2164-11-296 20459828 PMC2876125

[B56] Nadachowska-Brzyska K Zielinski P Radwan J Babik W Interspecific hybridization increases MHC class II diversity in two sister species of newts Mol Ecol 2012 21 887 906 10.1111/j.1365-294X.2011.05347.x 22066802

[B57] Babik W Durka W Radwan J Sequence diversity of the MHC DRB gene in the Eurasian beaver (*Castor fiber*) Mol Ecol 2005 14 4249 4257 10.1111/j.1365-294X.2005.02751.x 16313590

[B58] Babik W Methods for MHC genotyping in non-model vertebrates Mol. Ecol. Res 2010 10 237 251 10.1111/j.1755-0998.2009.02788.x 21565019

[B59] Balakrishnan CN Ekblom R Völker M Westerdahl H Godinez R Kotkiewicz H Burt DW Graves T Griffin DK Warren WC Edwards SV Gene duplication and fragmentation in the zebra finch major histocompatibility complex BMC Biol 2010 8 29 10.1186/1741-7007-8-29 20359332 PMC2907588

[B60] Radwan J Biedrzycka A Babik W Does reduced MHC diversity decrease viability of vertebrate populations? BiolConserv 2010 143 537 544 10.1016/j.biocon.2009.07.026 PMC7092871 32226082

[B61] Agudo R Alcaide M Rico C Lemus JA Blanco G Hiraldo F Donázar JA Major histocompatibility complex variation in insular populations of the Egyptian vulture: inferences about the roles of genetic drift and selection Mol Ecol 2011 20 2329 2340 10.1111/j.1365-294X.2011.05107.x 21535276

[B62] Lenz TB Becker S Simple approach to reduce PCR artefact formation leads to reliable genotyping of MHC and other highly polymorphic loci - implications for evolutionary analysis Gene 2008 427 117 123 10.1016/j.gene.2008.09.013 18848974

[B63] Librado P Rozas J DnaSP v5: a software for comprehensive analysis of DNA polymorphism data Bioinformatics 2009 25 1451 1452 10.1093/bioinformatics/btp187 19346325

[B64] Zhang Z Schwartz S Wagner L Miller W A greedy algorithm for aligning DNA sequences Journal Comput Biol 2000 7 203 214 10.1089/10665270050081478 10890397

[B65] Tamura K Dudley J Nei M Kumar S MEGA4: Molecular Evolutionary Genetics Analysis (MEGA) software version 4.0 Mol Biol Evol 2007 24 1596 1599 10.1093/molbev/msm092 17488738

[B66] Nei M Gojobori T Simple methods for estimating the numbers of synonymous and nonsynonymous nucleotide substitutions Mol Biol Evol 1986 3 418 426 3444411 10.1093/oxfordjournals.molbev.a040410

[B67] Yang ZH PAML 4: Phylogenetic analysis by maximum likelihood Mol Biol Evol 2007 24 1586 1591 10.1093/molbev/msm088 17483113

[B68] Yang ZH Wong WSW Nielsen R Bayes empirical Bayes inference of amino acid sites under positive selection Mol Biol Evol 2005 22 1107 1118 10.1093/molbev/msi097 15689528

[B69] Yang Z Nielsen R Goldman N Pedersen A-MK Codon-substitution models for heterogeneous selection pressure at amino acid sites Genetics 2000 155 431 449 10790415 10.1093/genetics/155.1.431 PMC1461088

[B70] Ronquist F Huelsenbeck JP MrBayes 3: Bayesian phylogenetic inference under mixed models Bioinformatics 2003 19 1572 1574 10.1093/bioinformatics/btg180 12912839

[B71] Akaike H A new look at the statistical model identification IEEE T Automat Contr 1974 19 716 723 10.1109/TAC.1974.1100705

[B72] Posada D jModelTest: Phylogenetic model averaging MolBiolEvol 2008 25 1253 1256 10.1093/molbev/msn083 18397919

[B73] Posada D Selection of models of DNA evolution with jModelTest Meth Mol Biol 2009 537 93 112 10.1007/978-1-59745-251-9_5 19378141

[B74] Martin DP Williamson C Posada D RDP2: recombination detection and analysis from sequence alignments Bioinformatics 2005 21 260 262 10.1093/bioinformatics/bth490 15377507

[B75] Martin D Rybicki E Detection of recombination amongst aligned sequences Bioinformatics 2000 16 562 563 10.1093/bioinformatics/16.6.562 10980155

[B76] Padidam M Sawyer S Fauquet CM Possible emergence of new geminiviruses by frequent recombination Virology 1999 265 218 225 10.1006/viro.1999.0056 10600594

[B77] Martin DP Posada D Crandall KA Williamson C A modified bootscan algorithm for automated identification of recombinant sequences and recombination breakpoints AIDS ResHumRetrov 2005 21 98 102 10.1089/aid.2005.21.98 15665649

[B78] Maynard Smith J Analysing the mosaic structure of genes J Mol Evol 1992 34 126 129 1556748 10.1007/BF00182389

[B79] Posada D Crandall KA Evaluation of methods for detecting recombination from DNA sequences: Computer simulations PNAS 2001 98 13757 13762 10.1073/pnas.241370698 11717435 PMC61114

[B80] Boni MF Posada D Feldman MW An exact nonparametric method for inferring mosaic structure in sequence triplets Genetics 2007 176 1035 1047 17409078 10.1534/genetics.106.068874 PMC1894573

[B81] Rice WR Analyzing tables of statistical tests Evolution 1989 43 223 225 10.2307/2409177 28568501

