# Supplementary material for: Ancestral polymorphism at the major histocompatibility complex (MHCIIß) in the Nesospiza bunting species complex and its sister species (Rowettia goughensis)
Source: BMC Evol Biol. 2012 Aug 15;12:143. doi: 10.1186/1471-2148-12-143 (PMC3483275; doi:10.1186/1471-2148-12-143)
Supplement: Additional file 1 — Table S1. List of 23 major histocompatibility complex class II ß (MHCIIß) exon 2 Nesospiza bunting sequences used in the present study. [file 1471-2148-12-143-S1.docx]

**Supplementary Table 1. List of 23 major histocompatibility complex class II ß (MHCIIß) exon 2 *Nesospiza* bunting sequences used in the present study.**

>Neso01 [organism = Nesospiza sp.] MHC exon II B haplotype01

AAGGTGAAGTTCGTGCAGAGGAGCATCTACAACAGGGAGCAGTTCCTGATGTTCGACAACGACGTGGGGCACTTTGTGGGGTTCGCCCCCTATGGGGAGAGGAATGCCAAGCGCTGGAATAGCGACCCAAATCTTCTGGAGGTTTATCAGGCTGCGGTG

>Neso02 [organism = Nesospiza sp.] MHC exon II B haplotype02

AAGGTGAAGTTCGTGCAGAGGAGCATCTACAACAGGGAGCAGTTCCTGATGTTCGACAGCGACAGCGACGTGGGGCGCTTTGTGGGGTTCGCCCCCTACGGGGAGAGGAATGCCAAGCGCTGGAATAGCGACCCAAATCTTCTGGAGGTTTATCAGGCTGCGGTG

>Neso03 [organism = Nesospiza sp.] MHC exon II B haplotype03

AAGGTGAAGTTCGTGCAGAGGAGCATCTACAACAGGGAGCAGTTCCTGATGTTCGACAGCGACGTGGGGCACTCTGTGCGGTTCGCCCCCTATGGGGAGAGGGATGCCAAGCGCTGGAATAGCGACCCAAATCTTCTGGAGGTTTATCAGGCTGCGGTG

>Neso04 [organism = Nesospiza sp.] MHC exon II B haplotype04

AAGGTGAAGTTCGTGCAGAGGAGCATCTACAACAGGGAGCAGTTCCTGATGTTCGACAGCGACGTGGGGCACTTTGCGGGGTTCGCCCCCTACGGGGAGAGGAATGCCAAGCGCTGGAATAGCGACCCAAATCTTCTGGAGGTTTATCAGGCTGCGGTG

>Neso05 [organism = Nesospiza sp.] MHC exon II B haplotype05

AAGGTGAAGTTCGTGCAGAGGAGCATCTACAACAGGGAGCAGTTCCTGATGTTCGACAGCGACGTGGGGCACTTTGTGCGGTTCGCCCCCTATGGGGAGAGGGATGCCAAGCGCTGGAATAGCGACCCAAATCTTCTGGAGGTTTATCAGGCTGCGGTG

>Neso06 [organism = Nesospiza sp.] MHC exon II B haplotype06

AAGGTGAAGTTCGTGCAGAGGAGCATCTACAACAGGGAGCAGTTCCTGATGTTCGACAGCGACGTGGGGCACTTTGTGGAGATTGCCCCCTATGGGGAGAGGAATGCCAAGCGCTGGAATAGCGACCCAAATCTTCTGGAGGTTTATCAGGCTGCGGTG

>Neso07 [organism = Nesospiza sp.] MHC exon II B haplotype07

AAGGTGAAGTTCGTGCAGAGGAGCATCTACAACAGGGAGCAGTTCCTGATGTTCGACAGCGACGTGGGGCACTTTGTGGGGTTCGCCCCCTACGGGGAGAGGAATGCCAAGCTCTGGAATAGCGACCCAAATCTTCTGGAGGTTTATCAGGCTGCGGTG

>Neso08 [organism = Nesospiza sp.] MHC exon II B haplotype08

AAGGTGAAGTTCGTGCAGAGGAGCATCTACAACAGGGAGCAGTTCCTGATGTTCGACAGCGACGTGGGGCACTTTGTGGGGTTCGCCCCCTATGGGGAGAGGAATGCCAAGCGCTGGAATAGCGACCCAAATCCTCTGGAGGTTTATCAGGCTGCGGTG

>Neso09 [organism = Nesospiza sp.] MHC exon II B haplotype09

AAGGTGAAGTTCGTGCAGAGGAGCATCTACAACAGGGAGCAGTTCCTGATGTTCGACAGCGACGTGGGGCACTTTGTGGGGTTCGCCCTCTACGGGGAGAGGAATGCCAAGCGCTGGAATAGCGGCCCAAATCCTCTGGAGGTTTATCAGGCTGCGGTG

>Neso10 [organism = Nesospiza sp.] MHC exon II B haplotype10

AAGGTGAAGTTCGTGCAGAGGAGCATCTACAACAGGGAGCAGTTCCTGATGTTCGACAGCGACGTGGGGCACTTTGTGGGGTTTGCCCCCTATGGGGAGAGGAATGCCAAGCGCTGGAATAGCGACCCAAATCTTCTGGAGGTTTATCAGGCTGCGGTG

>Neso11 [organism = Nesospiza sp.] MHC exon II B haplotype11

AAGGTGAAGTTCGTGCAGAGGAGCATCTACAACAGGGAGCAGTTCCTGATGTTCGACAGCGACGTGGGGCGCTTTGTGGGGTTCGCCCCCTACGGGGAGAGGAATGCCAAGCGCTGGAATAGCGACCCAAATCTTCTGGAGGTTTATCAGGCTGCGGTG

>Neso12 [organism = Nesospiza sp.] MHC exon II B haplotype12

AAGGTGAAGTTCGTGCAGAGGAGCATCTACAACAGGGAGCAGTTCCTGATGTTCGACAGCGACTTGGGGCACTTTGTGGGGTTTGCCCCCTATGGGGAGAGGAATGCCAAGCGCTGGAATAGCGACCCAAATCTTCTGGAGGTTTATCAGGCTGCGGTG

>Neso13 [organism = Nesospiza sp.] MHC exon II B haplotype13

AAGGTGAAGTTCGTGCAGAGGAGCATCTACAACAGGGAGCAGTTCCTGATGTTGGACAGCGACGTGGGGCACTTTGTGGGGTTCGCCCCCTACGGGGAGAGGAATGCCAAGCGCTGGAATCGCGACCCAAATCTTCTGGAGGTTTATCAGGCTGCGGTG

>Neso14 [organism = Nesospiza sp.] MHC exon II B haplotype14

AAGGTGAGGCTCGTGGAGAGGTACATCTACAACCGGCAGCAGCACGCGATGTTCGACAGCGACGTGGGGCGCTACGTGGGGTTCACCCCCTTCGGGGAGAAACAGGCCCAGTACTGGAACAGCAACCCGGAAATCATGGAGCGGAAACGGGCTGAGGTG

>Neso15 [organism = Nesospiza sp.] MHC exon II B haplotype15

AAGGTGAGGCTCGTGGAGAGGTACATCTACAACCGGCAGCAGCACGCGATGTTCGACAGCGACGTGGGGCGCTACGTGGGGTTCACCCCCTTTGGGGAGAAACAGGCCCAGTACTGGAACAGCAACCCGGAAATCATGGAGCGGAAACGGGCTGAGGTG

>Neso16 [organism = Nesospiza sp.] MHC exon II B haplotype16

AAGGTGAGGCTCGTGGAGAGGTACATCTACAACCGGCAGCAGCTCGCGATGTTCGACAGCGACGTGGGGCGCTACGTGGGGTTCACCCCCTTTGGGGAGAAACAGGCCCAGTACTGGAACAGCAACCCGGAAATCATGGAGCGGAAACGGGCTGAGGTG

>Neso17 [organism = Nesospiza sp.] MHC exon II B haplotype17

AAGGTGAGGCTCGTGGAGAGGTACATCTACAACCGGCAGCAGTACGCGATGTTCGACAGCGACGTGGGGCGCTACGTGGGGTTCACCCCCTTTGGGGAGAAACAGGCCCAGTACTGGAACAGCAACCCGGAAATCATGGAGCGGAAACGGGCTGAGGTG

>Neso18 [organism = Nesospiza sp.] MHC exon II B haplotype18

AAGGTGAGGCTCGTGGAGAGGTGCATCTACAACCGGCAGCAGCACGCGATGTTCGACAGCGACGTGGGGCGCTACGTGGGGTTCACCCCCTTTGGGGAGAAACAGGCCCAGTACTGGAACAGCAACCCGGAAATCATGGAGCGGAAACGGGCTGAGGTG

>Neso19 [organism = Nesospiza sp.] MHC exon II B haplotype19

AAGGTGAGGTTCGTGGACAGGTACATCTACAACCGGCAGCAGTACGCGATGTTCGACAGCGACGTGGGGCACTACGTGGGGTTCACCCCCTTTGGGGAGAGGGTGGCCAAGTACTGGAACAGCGACCCGGAAATCATGGAGCGGAAACGGGCTGCGGTG

>Neso20 [organism = Nesospiza sp.] MHC exon II B haplotype20

ACGGTGAAGTTCGTGCAGAGGAGCATCTACAACAGGGAGCAGTTCCTGATGTTCGACAGCGACGTGGGGCACTTTGTGGGGTTCGCCCCCTATGGGGAGAGGAATGCCAAGCGCTGGAATAGCGACCCAAATCTTCTGGAGGTTTATCAGGCTGCGGTG

>Neso21 [organism = Nesospiza sp.] MHC exon II B haplotype21

ACGGTGAAGTTCGTGCAGAGGAGCATCTACAACAGGGAGCAGTTCCTGATGTTCGACAGCGACGTGGGGCACTTTTTGGGGTTTGCCCCCTATGGGGAGAGGAATGCCAAGCGCTGGAATAGCGACCCAAATCTTCTGGAGGTTTATCAGGCTGCGGTG

>Neso22 [organism = Nesospiza sp.] MHC exon II B haplotype22

AGGGTGCAGTACGTGGAGAGGCACAGCTACAACCGGGAGGAGATCCTGCACTTCGACTGCGACGTGGGGCACTTTGTGGGGTTCACCCGCTTTGGGGAGAAGGTGGCCGGGTACTGGAACAGCCTCCCAGATTTCATGCGGCTAAAAAGGACTGCGGTG

>Neso23 [organism = Nesospiza sp.] MHC exon II B haplotype23

TGGGTGCAGTACGTAGAGAGGTACAGCTACAACCGGGAGGAGATCCTGCACTTCGACTGCGACGTGGGGCACTTTGTGGGGTTCACCCGCTTTGGGGAGAAGGTGGCCGGGTACTGGAACAGCCTCCCAGATTTCATGCGGCTAAAAAGGACTGCGGTG
